# A novel t(3;12)(q21;p13) translocation in a patient with accelerated chronic myeloid leukemia after imatinib and nilotinib therapy

**DOI:** 10.7497/j.issn.2095-3941.2013.01.008

**Published:** 2013-03

**Authors:** Ayda Bennour, Ikram Tabka, Yosra Ben Youssef, Zahra Kmeira, Abderrahim Khelif, Ali Saad, Halima Sennana

**Affiliations:** 1 Department of Cytogenetics, Molecular Genetics and Reproductive Biology, Farhat Hached University Teaching Hospital, Sousse 4000, Tunisia;; 2 Department of Clinical Hematology, Farhat Hached Hospital, Sousse 4000, Tunisia

**Keywords:** Philadelphia chromosome, t(3, 12)(q21, p13), chronic myeloid leukemia, accelerated phase, fluorescence in situ hybridization

## Abstract

The acquisition of secondary chromosomal aberrations in chronic myeloid leukemia (CML) patients with Philadelphia chromosome-positive (Ph+) karyotype signifies clonal evolution associated with the progression of the disease to its accelerated or blastic phase. Therefore, these aberrations have clinical and biological significance. T(3;12)(q26;p13), which is a recurrent chromosomal aberration observed in myeloid malignancies, is typically associated with dysplasia of megakaryocytes, multilineage involvement, short duration of any blastic phase, and extremely poor prognosis. We have identified a recurrent reciprocal translocation between chromosomes 3 and 12 with different breakpoint at bands 3q21 in the malignant cells from a 28-year-old man. The patient was initially diagnosed as having Ph+ CML in the chronic phase. The t(3;12)(q21;p13) translocation occurred 4 years after the patient was first diagnosed with CML while undergoing tyrosine kinase inhibitor therapy. We confirmed the t(3;12)(q21;p13) translocation via fluorescence in situ hybridization assay by using whole-chromosome paint probes for chromosomes 3 and 12. Our findings demonstrate that, similar to other recurrent translocations involving 3q26 such as t(3;3) and t(3;21), the t(3;12)(q21;p13) translocation is implicated not only in myelodysplastic syndrome and acute myeloid leukemia but also in the progression of CML. These findings extend the disease spectrum of this cytogenetic aberration.

## Introduction

Chronic myeloid leukemia (CML) is a clonal myeloproliferative disorder of the pluripotent hematopoietic progenitor cells and is characterized by massive proliferation and accumulation of myeloid cells that differentiate normally^1^^,^^2^. The cytogenetic hallmark of CML is the Philadelphia (Ph) chromosome that results from a reciprocal t(9;22)(q34;q11.2) translocation or its variants t(V;9;22)^3^. The molecular consequence of the t(9;22)(q34;q11.2) translocation is the formation of the BCR/ABL fusion gene, which is usually located on the Ph chromosome^4^. Clinically, CML progresses through three distinct phases: the easily controlled chronic phase, the ill-defined unstable accelerated phase, and the terminal blastic phase^5^.

The latter phase resembles acute leukemia and is highly refractory to chemotherapy with ≤20% response rate and a median survival period of 3 months to 6 months^5^^,^^6^. Majority of the CML patients showed changes only in the Ph chromosome throughout the chronic phase. As CML progresses to the advanced phases, the Ph chromosome-positive (Ph+) cells acquire new karyotypic abnormalities, most often a second copy of the Ph chromosome, i(17q), and trisomy 8 and 19. The increased genetic instability of the Ph+ leukemic clone facilitates the emergence of subclones with highly malignant phenotypes^6^. In this paper, we report a patient with Ph+ CML who acquired a novel additional chromosomal aberration t(3;12)(q21;p13) during the course of disease progression. This aberration was confirmed with fluorescence in situ hybridization (FISH).

## Case report

In October 2005, a 28-year-old man was referred to our hospital because of leukocytosis, splenomegaly, and weight loss. Peripheral blood count showed hemoglobin: 9.4 g/dL; platelets: 253×10^9^/L; white blood cells (WBC): 292×10^9^/L with 32% neutrophils, 2% lymphocytes, 3% eosinophils, 3% basophils, 58% immature granulocytes, and 2% blasts. Myeloid hyperplasia of the bone marrow (BM) with high proportion of cells at different stages of maturation, moderate eosinophilia without an excess of blasts, and 3% blasts was found. Cytogenetic analyses were performed on BM cells. Metaphases were obtained after short-term unstimulated cultures (24 and 48 h). The chromosomes were identified by heating R-bands through the Giemsa (RHG) technique. The chromosomes were classified following the Guidelines for the International System for Human Nomenclature^7^. FISH was performed by using whole-chromosome paint probes for chromosomes 3 (spectrum green)/12 (spectrum orange) (Vysis, Inc., Downers Grove, IL, USA). All of the details concerning the pretreatment of slides and hybridization were carried out according to the instructions accompanying the probes. The chromosomes were counterstained with 4’,6-diamidino-2-phenylindole and viewed with an Axioskop fluorescence microscope (Zeiss, Germany) by using triple excitation/emission bandpass filters. At least 10 metaphase cells were analyzed.

Chromosomal analysis revealed 46,XY, t(9;22)(q34;q11). The patient was diagnosed as having CML in the chronic phase. Treatment was initiated with hydroxyurea (1,500 mg daily dose). Eight months after the treatment, the WBC of the patient decreased to 4.7×10^9^/L (with 43% neutrophils), platelets: 132×10^9^/L; hemoglobin: 14 g/dL. The patient was then treated with imatinib mesylate (400 mg daily dose). After 6 months, cytogenetic analysis revealed a major cytogenetic response with 10% Ph+ cells. Twelve months after imatinib treatment initiation, a repeat R-banding karyotype showed a complete cytogenetic response (CCyR). After 24 months, the patient’s blood count showed WBC: 5.5×10^9^/L (65% neutrophils, 33% lymphocytes, 1% monocytes, and 1% basophils); hemoglobin: 13.4 g/dL; platelets: 177×10^9^/L. Karyotype analysis showed additional chromosomal abnormality (trisomy 8): 47,XY,+8,t(9;22)(q34;q11)/46,XY. Subsequently, the dose of imatinib was raised to 600 mg daily. After 6 months, CCyR was reached again.

After 24 months, the patient’s blood count showed WBC: 5.3×10^9^/L (58% neutrophils, 36% lymphocytes, 5% monocytes, and 0.5% eosinophils); hemoglobin: 11.5 g/dL; platelets: 162×10^9^/L. Cytogenetic analysis revealed two cell clones with additional chromosomal abnormalities: 47, XY, t(9;22)(q34;q11),+der(22)t(9;22)/48, idem, +8/46, XY.

Treatment with nilotinib (200 mg daily) was initiated, and 2 months later, the blood count showed WBC: 6.7×10^9^/L (66% neutrophils, 26% lymphocytes, 5% monocytes, and 0.5% eosinophils); hemoglobin: 14.2 g/dL; platelets: 219×10^9^/L.

CCyR and complete molecular response were reached throughout 3 months until the patient discontinued nilotinib treatment. Hematologic parameters showed that hemoglobin was 13.8 g/dL, platelets were 273×10^9^/L, and WBC was 60.3×10^9^/L (61% neutrophils, 7% lymphocytes, 4% monocytes, 1% erythroblasts, 20% immature granulocytes, and 7% blastic cells) (**Table 1**).

**Table 1:** Clinical data of patient

Time from diagnosis	Treatment	Hematologic parameters			Karyotype
		hb g/dL	P×10^9^/L	WBC×10^9^/L	
Diagnosis		9.4	253	292	46,XY, t(9;22)(q34;q11)
8 months	Hydroxyurea	14	132	4.7	46,XY, t(9;22)(q34;q11)
20 months	Imatinib 400 mg	—	—	—	46,XY
24 months		13.4	177	5.5	47,XY,+8,t(9;22)(q34;q11) /46,XY
30 months	Imatinib 600 mg	—	—	—	46,XY
54 months	—	11.5	162	5.3	47,XY,t(9;22)(q34;q11),+der(22)t(9;22)/48,idem,+8/46,XY
56 months	Nilotinib 200 mg	14.2	219	6.7	
59 months	Nilotinib 200 mg	13.8	273	60.3	46,XY
65 months	—	—	—	—	46,XY,t(3;12)(q21;p13), t(9;22)(q34;q11)/46,XY

Hb, hemoglobin; P, platelets; WBC, white blood cells.

**Figure 1:**
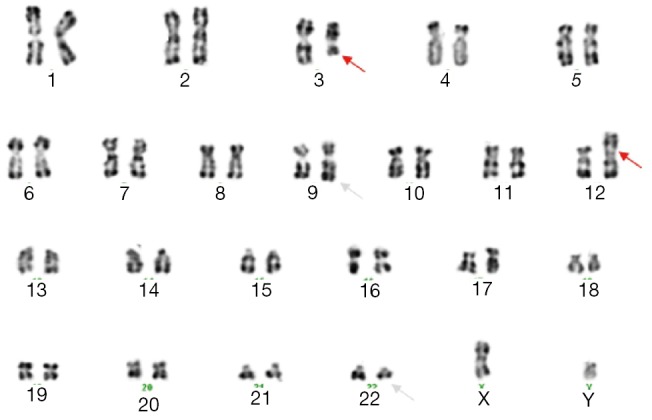
Karyotype from metaphase cell of the patient showing 46,XY,t(3:12)(q21;p13), t(9;22)(q34;q11). Chromosomes were identified by using RHG banding. The arrowheads indicate the location of the breakpoint on derivative chromosomes.

**Figure 2:**
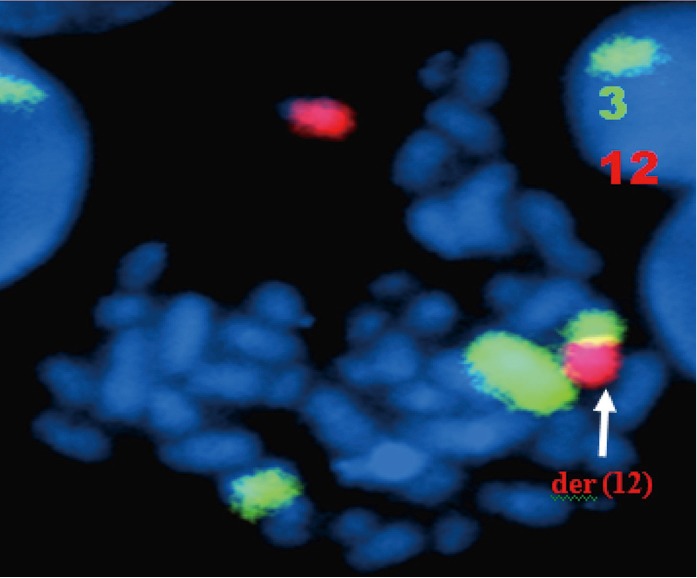
FISH analysis of the metaphase cell of the patient: chromosome painting [chromosomes 3 (green) and 12 (red)] displays an insertion of chromosome 3 material into chromosome 12.

Bone marrow aspiration concluded to an accelerated phase with 14% blastic cells (17% promyelocytes, 34% neutrophils, 20% eosinophils, and 6% erythroblasts) as well as an average bone marrow cellularity with rare megakaryocytes, platelets, and 21% myelocytes and metamyelocytes. Conventional karyotype analysis revealed additional recurrent translocation with t(9;22):46,XY,t(3;12)(q21;p13), t(9;22)(q34;q11) (**Figure 1**), which was confirmed with FISH by using whole-chromosome paint probes for chromosomes 3 and 12 (**Figure 2**).

## Discussion

We report a cytogenetically identical t(3;12)(q21;p13) translocation in one patient with accelerated CML. To our knowledge, the t(3;12)(q21;p13) translocation has not yet been described in the literature in patients with CML^8^. However, two cases of refractory anemia with t(3;12)(q21;p13) have been reported^9^^,^^10^.

All attempts to induce remission were unsuccessful, which suggests that this chromosomal abnormality may be associated with poor prognosis.

Structural abnormalities affecting the long arm of chromosome 3, mostly including paracentric inversions inv(3)(q21q26), insertions ins(3)(q2lq25q26), homologous translocations t(3;3)(q2;q26), and translocations between 3q26, and other chromosomes such as t(3;21)(q26;q22) and t(2;3)(q21;q26)(5;7;10), have been described in 140 cases^11^^-^^13^. These specific chromosomal rearrangements have been demonstrated in all French–American–British subtypes of acute myeloid leukemia except M3. These chromosomal rearrangements have also been shown in rare cases of myelodysplastic syndrome (MDS), and in the blastic crisis of CML (CML-BC)^14^.

The t(3;12)(q26;p13) translocation has recently been reported as a new recurrent translocation in MDS and CML-BC^15^. This translocation involves the ETV6 gene (formerly named TEL), a new member of the ETS gene family of transcription factors at 12p13 and the EVI1 (zinc finger transcription factor) gene at 3q26. This translocation, which also generates the TEL/EVI1 fusion gene, could lead to the inappropriate expression of EVI1 protein driven by the TEL promoter and could contribute to malignant transformation^15^. The t(3;12) translocation involving 3q21 breakpoint has never been reported in CML until that time^8^.

The incidence of 3q anomalies with breakpoints in q21 and q26 is shown to be higher than previously estimated. These anomalies can be considered as frequent and specific chromosomal changes in myeloid malignancies^11^^,^^16^. The breakpoints in both 3q regions are scattered over considerable distances^17^^,^^18^.

Thus, similar to the t(3;12)(q21;pl3) translocation, the t(3;12)(q26;pl3) translocation appears as a rare but nonrandom event observed in various myeloid leukemia subtypes, which are frequently associated with dysplasia of megakaryocytes, multilineage involvement, short duration of any blastic phase, and extremely poor prognosis.

In contrast to 3q26, the precise role of the sequences in 3q21 is uncommon and less well described. The breakpoints are clustered, and the 10 breakpoints that have been mapped are located within a segment no more than 30 kb in size^19^^-^^21^. However, no candidate genes for leukemia have been described.

The most intriguing question generated by this study is whether the formation of t(3;12)(q21;p13) in the t(9;22)(q34;q11) positive clone is related to therapy or to the accelerated phase of CML.

The co-existence of t(9;22) and t(3;26) has been reported in only one patient with CML^22^. The t(3;12)(q26;p13) translocation emerged as a sole additional chromosomal abnormality 16 months prior to the blastic crisis. The patient received combination chemotherapy with cytosine arabinoside and daunorubicin. The patient achieved complete remission after bone marrow transplantation^22^.

The patient received tyrosine kinase inhibitor (TKI) therapy for a total of 4 years. However, this dose of TKI therapy is most likely related to the development of t(3;12).

Therefore, we can speculate that the acquisition of trisomy 8 and the additional Ph chromosome followed by the acquisition of t(3;12) in this case is either a result of the natural history of the CML characterized initially by t(9;22) and +8 or reflects the simultaneous development of two clonal myeloid populations.

The puzzling but intriguing role of trisomy 8 in the disease progression in these patients remains uncertain. The trisomy 8 in this case may represent either the t(3;12) clone or the Ph+ clone that developed simultaneously.

The hypothesis may not be consistent with the current dogma; 100% of BCR-ABL1 + cells bear concomitant trisomy 8 cells, which is thought to be associated with an accelerated phase although blast cells were not found and the patient has a WBC of 7.2×10^9^/L.

We conclude that some patients with CML are either in the accelerated phase with coexistence of t(9;22) and t(3;12) with or without trisomy 8 or would advance to blast crisis and simultaneously carry BCR-ABL and duplicate Ph, trisomy 8, or other cytogenetic abnormalities, such as the t(3;12) detected in this case. This progression may occur after hydroxyurea or TKI therapy.

## Conclusions

We have described a patient with a novel unbalanced t(3;21)(q26;q22) translocation emerging with t(9;22) in the accelerated phase of CML. To our knowledge, this additional chromosomal aberration that involves breakpoint at 3q21 has not yet been described. This aberration is thought to play a crucial role in CML progression.

Results show that additional cytogenetic abnormalities are frequently detected and are significantly associated with poor overall survival in patients with Ph+ CML after failure of imatinib treatment. This finding indicates that conventional cytogenetics on metaphases remain mandatory at diagnosis and during follow-up and should not be replaced by techniques that analyze only BCR-ABL.

The decreased efficacy of nilotinib in the context of additional cytogenetic abnormalities may reflect intrinsic aggressiveness of the disease, thus prompting the close monitoring of this disease.

## References

[1] Deininger MW Goldman JM Chronic myeloid leukemia. Curr Opin Hematol 1998;5:302-308 9747637 10.1097/00062752-199807000-00010

[2] Sawyers CL Chronic myeloid leukemia. N Engl J Med 1999;340:1330-1340 10219069 10.1056/NEJM199904293401706

[3] Rowley J. A new consistent chromosomal abnormality in chronic myelogenous leukemia identified by quinacrine fluorescence and Giemsa staining. Nature 1973;43:290-293 4126434 10.1038/243290a0

[4] Deininger MW Goldman J Melo J. The molecular biology of chronic myeloid leukemia. Blood 2000;96:3343-3356 11071626

[5] Sacchi S Kantarjian H O’Brian S Cortes J Rios MB Giles FJ Chronic myelogenous leukemia in nonlymphoid blastic phase: analysis of the results of first salvage therapy with three different treatment approaches for 162 patients. Cancer 1999;86:2632-2641 10594858 10.1002/(sici)1097-0142(19991215)86:12<2632::aid-cncr7>3.0.co;2-a

[6] Bernstein R. Cytogenetics of chronic myelogenous leukemia. Semin Hematol 1988;25:20-34 3279513

[7] ISCN (2009): An international system for human cytogenetic nomenclature. In: ShaVer LG, Tommerup N (eds) Karger.

[8] Mitelman F, Johansson B, Mertens F(Eds.) Mitelman Database of Chromosome Aberrations in Cancer (2012). http://cgap.nci.nih.gov/Chromosomes/Mitelman

[9] Fleischman EW Reshmi S Sokova OI Kirichenko OP Konstantinova LN Kulagina OE Increased karyotype precision using fluorescence in situ hybridization and spectral karyotyping in patients with myeloid malignancies. Cancer Genet Cytogenet 1999;108:166-170 9973948 10.1016/s0165-4608(98)00137-x

[10] Mauritzson N Johansson B Rylander L Albin M Strömberg U Billström R The prognostic impact of karyotypic subgroups in myelodysplastic syndromes is strongly modified by sex. Br J Haematol 2001;113:347-356 11380398 10.1046/j.1365-2141.2001.02750.x

[11] Fonatsch C Gudat H Lengfelder E Wandt H Silling-Engelhardt G Ludwig WD Correlation of cytogenetic findings with clinical features in 18 patients with inv(3)(q21q26) or t(3;3)(q21;q26). Leukemia 1994;8:1318-1326 8057667

[12] Horsman DE Gascoyne RD Barnett M Acute leukemia with structural rearrangements of chromosome 3. Leukemia Lymphoma 1995;16:369-377 7787746 10.3109/10428199509054422

[13] Secker-Walker LM Mehtia A Bain B Abnormalities of 3q21 and 3q26 in myeloid malignancy: a United Kingdom Cancer Cytogenetic Group study. Br J Haematol 1995;91:490-501 8547101 10.1111/j.1365-2141.1995.tb05329.x

[14] Heim S, Mitelman F. Cancer Cytogenetics. New York: Alan R. Liss, Inc, 1987:65-128.

[15] Raynaud SD Baens M Grosgeorge J Rodgers K Reid CD Dainton M Fluorescence in situ hybridization analysis of t(3;12)(q26;p13): a recurring chromosomal abnormality involving the TEL gene (ETV6) in myelodysplastic syndromes. Blood 1996;88:682-689 8695816

[16] Bellomo MJ Parlier V Muhlematter D Grob JP Beris P Three new cases of chromosome 3 rearrangements in band q21 and q26 with abnormal thrombopoiesis bring further evidence to the existence of a 3q213q26 syndrome. Cancer Genet Cytogenet 1992;59:138 1581880 10.1016/0165-4608(92)90208-p

[17] Morishita K Parganas E Willman CL Whittaker MH Drabkin H Oval J Activation of the EVI1 gene expression in human acute myelogenous leukemias by translocations spanning 300–400 kilobases on chromosome 3q26. Proc Natl Acad Sci 1992;89:3937-3941 1570317 10.1073/pnas.89.9.3937 PMC525606

[18] Levy ER Parganas E Morishita K Fichelson S James L Oscier D DNA rearrangements proximal to the EVI1 locus associated with the 3q21q26 syndrome. Blood 1994;83:1348-1354 8118036

[19] Suzukawa K Parganas E Gajjar A Abe T Takahashi S Tani K Identification of a breakpoint cluster region 3’ of the Ribophorin I gene at 3q21 associated with the transcriptional activation of the EVIl gene in acute myelogenous leukemia with inv(3)(q21q26). Blood 1994;84:2681-2688 7919381

[20] Pekarsky Y Zabarovsky E Kashuba V Drabkin H Sandberg AA Morgan R Cloning of breakpoints in 3q21 associated with hematologic malignancy. Cancer Genet Cytogenet 1995;80:1-8 7697625 10.1016/0165-4608(94)00168-b

[21] Rynditch A Pekarsky Y Schnittger S Gardiner K. Leukemia breakpoint region in 3q21 is gene rich. Gene 1997;193:49-57 9249066 10.1016/s0378-1119(97)00076-0

[22] Nakamura Y Nakazato H Sato Y Furusawa S Mitani K. Expression of the TEL/EVI1 fusion transcript in a patient with chronic myelogenous leukemia with t(3;12)(q26;p13). Am J Hematol 2002;69:80-82 11835339 10.1002/ajh.10028

